# Cytokine Autoantibodies Are Associated with Infection Risk and Self-Perceived Health: Results from the Danish Blood Donor Study

**DOI:** 10.1007/s10875-020-00744-3

**Published:** 2020-01-15

**Authors:** Jakob H. von Stemann, Ole B. Pedersen, Henrik Hjalgrim, Christian Erikstrup, Henrik Ullum, Lise W. Thørner, Margit AH. Larsen, Kristoffer S. Burgdorf, Erik Sørensen, Morten B. Hansen, Sisse R. Ostrowski

**Affiliations:** 1 Department of Clinical Immunology, Rigshospitalet, Copenhagen University Hospital, Blegdamsvej 9, 2100 Copenhagen, Denmark; 2 Department of Clinical Immunology, Næstved Sygehus, Næstved, Denmark; 3 Epidemiology Research, Statens Serum Institut, Copenhagen, Denmark; 4 Department of Hematology, Rigshospitalet, Copenhagen University Hospital, Copenhagen, Denmark; 5 Department of Clinical Immunology, Aarhus University Hospital, Aarhus, Denmark

**Keywords:** Autoimmunity, cytokine autoantibodies, IL-10, IFNα, blood donors, epidemiology

## Abstract

The presence of naturally occurring cytokine-specific autoantibodies (c-aAb) in humans is well established, as well as associations to selected pathologies. However, the overall influence of c-aAb on immunocompetence remains largely unknown. In this paper, we performed a large-scale investigation of c-aAb association with infection risk. A cohort of healthy Danish blood donors was screened for c-aAb against IL-1α, IL-6, IL-10, IFNα, and GM-CSF using a Luminex-based multiplex assay, and results were linked to data from the Danish National Prescription Registry. The filing of an antimicrobial prescription following c-aAb measurement was used as a proxy for impaired immunocompetence. We found that c-aAb against pro-inflammatory cytokines IFNα and GM-CSF tended to associate with increased risk of prescription filings in women, whereas antibodies against anti-inflammatory IL-10 were associated with a lower predicted risk of antimicrobial prescriptions, as well as higher self-perceived health scores. We also observed an association of cumulative c-aAb presence with prescription risk. Our data show that cytokine autoantibodies in healthy individuals associate with various proxies for immunomodulation, with the exact association dependent on the pattern of pro- or anti-inflammatory cytokines targeted. This suggests that c-aAb may express cytokine-modulatory properties in healthy individuals and may be critical to further investigate as biomarkers of immunodeficiency.

**Electronic supplementary material:**

The online version of this article (10.1007/s10875-020-00744-3) contains supplementary material, which is available to authorized users.

## Introduction

Cytokines are pivotal signaling molecules in the immune system balancing pro- and anti-inflammatory immune responses [1]. There is emerging evidence that naturally occurring cytokine-specific autoantibodies (c-aAb) may critically influence immune competence [2–5].

The influence of c-aAb on the systemic cytokine networks remains mostly unknown, though multiple studies have linked specific c-aAb to various pathologies and opportunistic infections [6–8]. Most notably, granulocyte-macrophage colony-stimulating factor (GM-CSF) c-aAb have been identified as a causal factor for pulmonary alveolar proteinosis (PAP), a rare and debilitating respiratory disease [9, 10]. A range of c-aAb have also been labeled as phenotypic classifiers for various primary immunodeficiencies (including GM-CSF c-aAb for PAP) by the international union of immunological societies [11]. Conversely, c-aAb against pro-inflammatory interleukins (IL) have been associated with improved prognosis or reduced symptoms in some autoimmune diseases, such as rheumatoid arthritis and Sjögrens syndrome indicating that ultimately the biologic effect of c-aAb depends on the specific pathological context combined with the c-aAb specificity [12, 13].

The trigger for c-aAb production is debated. Some studies indicate that the genetic framework may promote c-aAb (e.g., specific variants of the antigen-presenting human leukocyte antigen or mutations of the autoimmune regulator gene, AIRE), in addition to microbe-induced loss of tolerance, through mechanisms such as molecular mimicry [14, 15].

We have previously reported that detectable levels of c-aAb are common among healthy individuals, with high levels being associated with older age and male sex. Furthermore, in healthy individuals, high levels of IL-6 c-aAb are associated with low C-reactive protein (CRP) levels, indicating a possible functional effect of naturally occurring, high-titer c-aAb [16].

The aim of the present study was to investigate the association between available indicators of c-aAb-mediated immune modulation, evidenced by infectious risk and self-perceived health. We hypothesized that high-titer levels of c-aAb may inflict immunomodulation to a degree that would either increase or reduce infectious risk and self-perceived health. Thus, we linked our previously c-aAb screened cohort of healthy blood donors to health register and questionnaire data to assess possible outcomes of c-aAb-mediated immunomodulation, with antimicrobial prescription filings and self-perceived health as endpoints. To our knowledge, this is the largest study of the biologic and potentially immunomodulatory effects of c-aAb in a healthy population to date.

## Materials and Methods

### Study Population

The study includes 8972 blood donors enrolled in 2010 in the first iteration of The Danish Blood Donor Study (DBDS), a nationwide prospective cohort study on blood donor health [17, 18]. All participants were healthy at the time of inclusion and between 18 and 67 years of age. At the day of inclusion, participants completed a written health questionnaire, and a plasma sample was retained for biomarker analysis, which included c-aAb measurements. Participants were censored from analysis in this study in the event of missing data for any dependent or independent variable.

### Coding of Dependent and Independent Variables

#### Epidemiological Covariates

Based on the questionnaire, information on potential covariates including participant body mass index (BMI), smoking status, and usage of combined oral contraceptives (OC) was obtained. BMI was calculated as self-reported weight (kg)/self-reported height (m) squared and was censored in the event of unrealistic anthropometric outliers (heights < 1,5 m and > 2,1 m, weight < 45 kg and > 160 kg). Participants were coded as smokers if they identified as currently smoking any number of cigarettes at the time of inclusion. Age was defined at sample date. Usage of OC was defined at the time of inclusion.

Charlson comorbidity score [19] was calculated based on the 15-year histories of selected diseases for participants, as previously described [20]. This was based on data from the Danish National Patient Registry, which records time, place, and primary/secondary diagnoses for all hospital visits in Denmark [21].

#### Cytokine Autoantibodies

C-aAb levels were measured as previously described [22], using plasma from participants collected at date of DBDS inclusion. Plasma was collected in 2010, and c-aAb levels were measured in 2014. High levels of c-aAb were defined as median fluorescence intensity (MFI) signals above the 99th percentile, to identify participants with c-aAb levels most likely to inhibit their target cytokines. Separate cutoff values were generated for men and women, due to differences in c-aAb signals between the groups, as previously described [16]. Low c-aAb levels were defined as MFI values below MFI + 4 standard deviations (SD) of the negative control, and intermediary c-aAb levels were defined as MFI values between the low and high MFI groups.

#### Antimicrobial Prescriptions

Data on prescription of antimicrobials were obtained from the Danish National Prescription Registry (DNPR), which records all pharmacy prescriptions of drugs in Denmark, from 1994 and onwards [23]. Prescriptions of antibacterials in general (atc = J01), tetracyclines (atc = J01A), penicillin (atc = J01C), sulfonamides (atc = J01E), macrolides (atc = J01F), antimycotics (atc = J02), and antivirals (atc = J05) were identified for the study participants. Follow-up began at the date of DBDS inclusion and ended at the first antimicrobial prescription being filled, censored at 1 year of follow-up. Follow-up was also censored in the event of death, emigration, or end of follow-up for prescription data (December 31, 2016). Depending on the type of prescription being analyzed, participant’s prescription history was defined as having had any prescriptions of the investigated antimicrobials or specific prescriptions of these, e.g., penicillin, macrolides, sulfonamides, etc., within 1 year prior to c-aAb measurement/DBDS inclusion.

#### Infection-Related Hospital Diagnoses

The Danish National Patient Registry was also used to identify diagnoses related to infection for the study participants, using the same ICD-10 criteria as previously, with chronic infections being excluded [20]. Briefly, the diagnoses investigated covered primary and secondary diagnoses and underlying medical conditions and included ICD-10 codes concerning infections. Follow-up time was defined as time between DBDS inclusion/c-aAb measurement and the first infection-related diagnosis within a year, censored like prescription data.

#### Self- Reported Health-Related Quality of Life Scores

The questionnaire included the short form 12 (SF-12) instrument, a validated form of the SF-36 instrument for estimation of health-related quality of life (HRQL), summarized as mental and physical component scores (MCS and PCS) [24]. MCS combines scales for vitality, social functioning, role-emotional, and mental health, and PCS combines physical functioning, role-physical, bodily pain, and general health. Lower scores correspond to poorer self-reported health, with mean values of 52.8 MCS (standard deviation (SD) = 8.3) and 51.0 PCS (SD = 8.1) in Danish individuals [25].

### Statistical Analyses

Due to previously described sex-specific variation regarding c-aAb distribution [16], descriptive data were stratified according to sex. Continuous normally distributed data were compared across sex by student’s t-test (c-aAb MFI signals were log-transformed, to approximate normal distributions).

To investigate the possible cytokine-inhibitory effects of high levels of c-aAb in healthy individuals, we applied a Cox proportional hazards model, with antimicrobial prescription and infectious diagnosis incidence as outcomes, as proxies for immune competence. Unadjusted and multivariate analyses adjusted for age, smoking, BMI, OC, and prescription history were conducted with high or intermediary levels of c-aAb as independent variables. Covariates for the multivariable analysis were selected based on associations with either prescriptions or diagnoses, with a *p* value of < 0.05 in univariate tests. The initial univariate tests included student’s t-tests to test for associations between c-aAb and antimicrobial prescriptions/diagnoses within 1 year following inclusion (coded as 0/1 for no or any prescription/diagnosis) with continuous variables (age, BMI, etc.) and chi-squared tests to test for associations between c-aAb and binary variables (smoking, sex, Charlson comorbidity score > 0, etc.). The outcomes in Cox analyses, i.e., filing antimicrobial prescriptions or infectious diagnoses, were assessed within 1 year of c-aAb measurement, using the low-level c-aAb group as reference (coded as 0 for low c-aAb and 1 for intermediary or high c-aAb levels, investigated separately). The investigated prescriptions were antibacterials in general (compound endpoint) and specifically penicillins, sulfonamides, macrolides, antivirals, tetracyclines, and antimycotics. These were the most common prescription categories within our dataset (data not shown).

T- and chi-squared tests were also used to investigate association between low, intermediary, and high c-aAb levels and the selected epidemiological covariates in the multivariate model. Participants with intermediary and high c-aAb levels were compared separately to participants with low c-aAb levels.

Association between C-aAb levels was investigated using Spearman’s rank correlation for nonparametric, continuous MFI signals, and chi-squared tests for associations between ordinal (coded as 2 = high, 1 = intermediary, and 0 = low levels of c-aAb) and binary (coded as 1 = high or 0 = non-high levels of c-aAb) variables.

In further multivariate Cox proportional hazards models, we analyzed the influence of combined c-aAb for infectious risk, by analyzing antimicrobial prescriptions stratified according to overlap between combinations of low, intermediary, and high levels of c-aAb. All possible c-aAb combinations were tested, two at a time (e.g., IL-1α and IL-6 c-aAb, IL-1α and IL-10 c-aAb, etc.), and were adjusted as described above. Participants with low levels of both investigated c-aAb were used as reference.

Logistic regressions were used to test for the predictive value of c-aAb (independent variables) for MCS or PCS (dependent variables), applying the same covariates as for the prescription/diagnosis analyses. Dichotomized variables representing low and high MCS and PCS were generated, based on continuous MCS/PCS scores below the 10th or above the 90th percentiles, respectively. Separate high/low MCS/PCS variables were generated for men and women and adjusted as described above in separate analyses.

All analyses were stratified by sex and performed using STATA (STATA/MP15 for PC, StataCorp, College Station, TX). *p* values below 0.05 were considered statistically significant. Data used for the analyses of this manuscript are not publicly available as they utilized the health registers of Statistics Denmark, which are restricted to Danish researches with explicit permits.

### Ethics

All participants provided oral and written informed consent at the time of inclusion. The study was approved by the Danish health research ethics committee system (RH30–4444 I-suite 00922), and the biobank and research database were approved by the Danish Data Protection Agency (P-2019-99).

## Results

### Characteristics of the Cohort

The sample population was 8972 participants from the Danish Blood Donor Study (DBDS) with measured levels of cytokine-specific autoantibodies (c-aAb) against IL-1α, IL-6, IL-10, interferon α (IFNα), and GM-CSF. A demographic overview of the participants with complete data for all variables (*n* = 8185) is shown in Table 1. All displayed variables were associated with antibacterial prescription filings in univariate tests. The population comprised 48.0% women and 52.0% men. Men were significantly older than women and had a significantly higher mental component score (MCS), IL-1α c-aAb, and IL-10 c-aAb, whereas women had higher levels of IL-6 c-aAb (*p* = 0.0016) and higher body mass index (BMI) and filled more antibacterial prescriptions within 1-year follow-up (all *p* < 0.0001). High levels of IL-1α, IL-6, and IL-10 c-aAb were positively associated with age, and high levels of IL-6 c-aAb were negatively associated with use of oral contraceptives (OC). These specific associations were not observed at the intermediary c-aAb level (Table S1). The degree of association between the five investigated c-aAb seemed to vary depending on their levels. When expressed as continuous variables of measured MFI and as ordinal variables grouped into high, intermediary, or low levels, positive correlations between all c-aAb were observed (Table S2). For associations between high levels of c-aAb, specifically a significant association was only observed for GM-CSF and IL-1α specific c-aAb in men (Table S2a).

**Table 1:** Characteristics of the study population

	Women	Men	*p* value ^a^
Number of participants	3926 (48.0%)	4259 (52.0%)	
Age (years) ^b^	38.5 (+ 12.3)	40.7 (+ 12.2)	< 0.0001
Smoking (current)	700 (17.8%)	698 (16.4%)	0.084
BMI	25.6 (+ 3.4)	24.5 (+ 4.1)	< 0.0001
Combined oral contraceptives (yes) ^c^	1086 (27.7%)	–	
MCS ^b^	52.1 (+ 6.7)	53.3 (+ 6.2)	< 0.0001
PCS ^b^	55.1 (+ 4.1)	55.1 (+ 3.9)	0.945
Filled at least one prescription for antibacterial ^d^	1425 (36.3%)	934 (21.9%)	< 0.0001
IL-1α c-aAb signal (MFI) ^b^	520 (+ 1282)	787 (+ 2037)	< 0.0001
Cutoff for high levels (MFI) ^e^	6909	11,765	
IL-6 c-aAb signal (MFI) ^b^	666 (+ 1633)	595 (+ 1656)	0.0016
Cutoff for high levels (MFI) ^e^	8007	6993	
IL-10 c-aAb signal (MFI) ^b^	129 (+ 387)	143 (+ 403)	< 0.0001
Cutoff for high levels (MFI) ^e^	881	1177	
IFNα c-aAb signal (MFI) ^b^	173 (+ 572)	169 (+ 521)	0.234
Cutoff for high levels (MFI) ^e^	2397	1799	
GM-CSF c- signal MFI ^b^	288 (+ 1312)	267 (+ 1150)	0.115
Cutoff for high levels (MFI) ^e^	5861	5287	

^a^Student’s t-test was used for comparison of continuous variables across sex (age, BMI, PCS, MCS, c-aAb levels), and chi-squared tests were used for comparison of binary variables (smoking, OC use, prescription history)

^b^Data presented as mean ± SD

^c^Data presented as percentage of population

^d^Data presented as incidence proportion, within 1 year of follow-up

^e^99th percentile of group MFI

### C-aAb and Predicted Risk of Antimicrobial Prescriptions

**Fig. 1:**
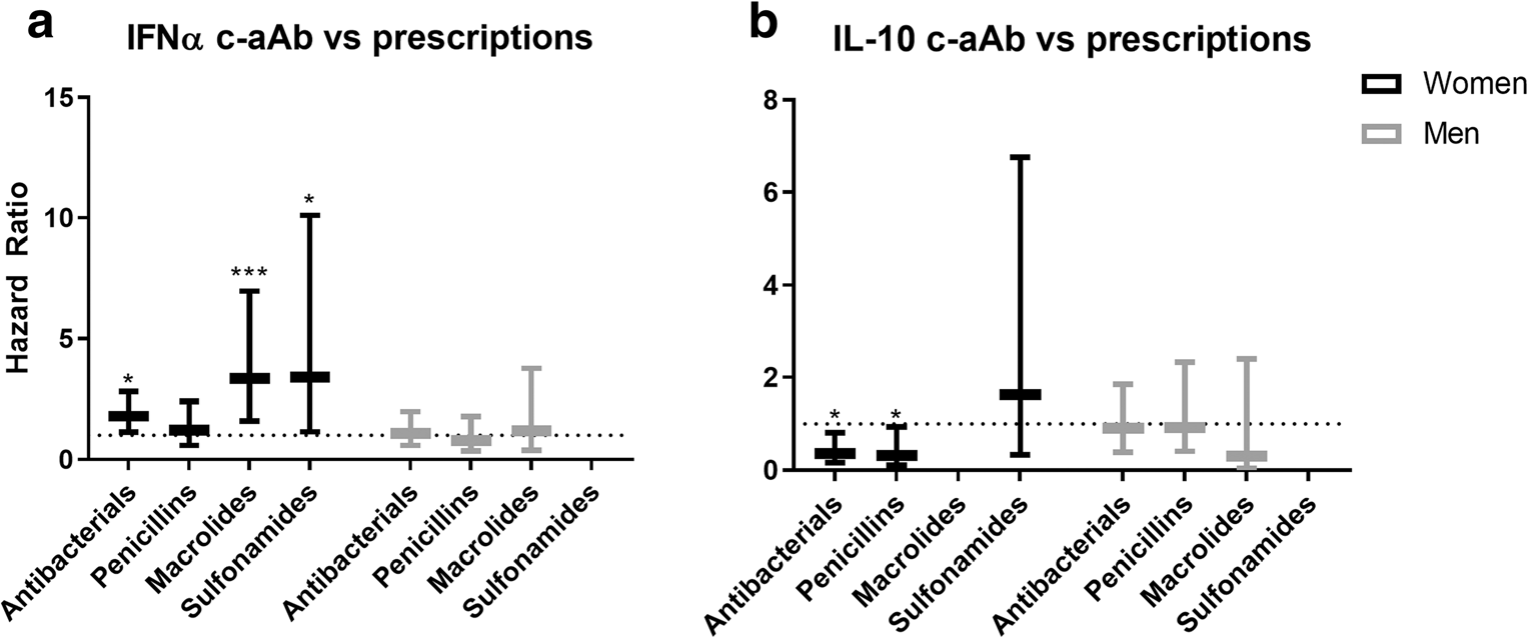
IFNα and IL-10 c-aAb as predictors of antimicrobial prescriptions Multivariate Cox proportional hazards models were used to investigate high levels of (**a**) IFNα c-aAb and (**b**) IL-10 c-aAb as predictors of risk of filing prescriptions for antibacterials in general and, specifically, for penicillins, macrolides, and sulfonamides within 1 year of c-aAb measurement. The models were adjusted for age, high levels of c-aAb, oral contraceptives, smoking, BMI, and 1 year prescription history of the prescription subtype in question prior to c-aAb measurement. For binary variables, high levels of c-aAb, usage of contraceptives, and having had at least one prescription a year before c-aAb measurement were coded as 1. High levels of c-aAb were defined as MFI above the 99th percentile, calculated separately for women and men. Data are presented as HR with 95% Confidence Interval (CI). * denotes a *p* value of < 0.05, *** *p* < 0.001

In the adjusted Cox proportional hazards models for women, high levels of IFNα c-aAb predicted increased risk of antibacterial prescriptions in general, as well as macrolide and sulfonamide prescriptions (Fig. 1A). GM-CSF c-aAb predicted increased risk of sulfonamide prescriptions (Table 2). In contrast, high levels of IL-10 c-aAb predicted reduced risk of antibacterial prescriptions in general and reduced risk of penicillin prescriptions (Table 2, Fig. 1B). These associations were observed with high levels of c-aAb, but not with intermediary levels.

**Table 2:** C-aAb as predictors of over-the-counter prescriptions within 1 year – women

C-aAb	Prescription	C-aAb level	IR ^a^	Crude HR (95%CI)	Adjusted HR (95%CI) ^b^	*p* value
IFNα	Antibacterials	Intermediary ^c^	1375/3002	1.00 (0.91–1.11)	1.01 (0.90–1.12)	0.982
		High^d^	780/1683	1.63 (1.05–2.44)	1.79 (1.13–2.81)	0.008
	Penicillins	Intermediary ^c^	1375/3335	0.98 (0.88–1.09)	0.97 (0.86–1.08)	0.552
		High^d^	516/1862	0.90 (0.47–1.73)	1.22 (0.58–2.41)	0.547
	Macrolides	Intermediary ^c^	267/3646	0.97 (0.77–1.21)	0.95 (0.74–1.22)	0.682
		High^d^	157/2043	3.60 (1.96–6.77)	3.61 (1.70–7.67)	0.001
	Sulfonamides	Intermediary ^c^	139/3714	1.16 (0.84–1.60)	1.27 (0.90–1.79)	0.171
		High^d^	73/2090	2.84 (1.05–7.94)	3.58 (1.19–10.64)	0.026
GM-CSF	Antibacterials	Intermediary ^c^	1315/2890	0.99 (0.86–1.17)	0.90 (0.80–1.18)	0.770
		High^d^	1277 /2767	1.44 (0.91–2.26)	1.57 (0.96–2.64)	0.070
	Penicillins	Intermediary ^c^	1315/3207	0.99 (0.84–1.17)	0.97 (0.81–1.18)	0.811
		High^d^	757/2895	1.24 (0.68–2.23)	1.32 (0.70–2.69)	0.353
	Macrolides	Intermediary ^c^	264/3500	1.09 (0.77–1.54)	1.01 (0.66–1.53)	0.949
		High^d^	238/3162	0.97 (0.31–3.02)	1.35 (0.35–4.01)	0.707
	Sulfonamides	Intermediary ^c^	135/3568	0.63 (0.34–1.18)	0.55 (0.26–1.16)	0.117
		High^d^	131/3217	2.74 (0.98–7.19)	3.21 (1.12–9.18)	0.029
IL-10	Antibacterials	Intermediary ^c^	1364/2954	1.02 (0.93–1.13)	0.99 (0.89–1.11)	0.839
		High^d^	827/1817	0.38 (0.18–0.81)	0.36 (0.16–0.81)	0.015
	Penicillins	Intermediary ^c^	1364/3282	1.01 (0.91–1.12)	0.98 (0.88–1.09)	0.697
		High^d^	522/1935	0.37 (0.14–0.97)	0.22 (0.05–0.89)	0.032
	Macrolides	Intermediary ^c^	271/3591	1.08 (0.86–1.34)	1.04 (0.82–1.33)	0.738
		High^d^	–	–	–	
	Sulfonamides	Intermediary ^c^	139/3663	1.06 (0.77–1.46)	1.05 (0.75–1.49)	0.768
		High^d^	79/2161	1.31 (0.33–5.30)	1.62 (0.33–6.76)	0.505
IL-6	Antibacterials	Intermediary ^c^	1358	0.86 (0.78–0.97)	0.84 (0.74–0.94)	0.003
		High^d^	475/938	0.79 (0.52–1.35)	0.73 (0.41–1.31)	0.301
	Penicillins	Intermediary ^c^	1358/3305	0.86 (0.78–0.97)	0.85 (0.76–0.95)	0.214
		High^d^	304/1053	0.62 (0.33–1.31)	0.49 (0.24–1.38)	0.119
	Macrolides	Intermediary ^c^	271/3609	0.96 (0.76–1.21)	0.90 (0.69–1.18)	0.440
		High^d^	92/1166	0.98 (0.32–2.98)	1.04 (0.33–3.39)	0.923
	Sulfonamides	Intermediary ^c^	141/3679	0.82 (0.59–1.14)	0.89 (0.63–1.28)	0.557
		High^d^	50/1187	0.56 (0.09–4.10)	0.80 (0.11–5.89)	0.824
IL-1α	Antibacterials	Intermediary ^c^	1378/2986	0.98 (0.89–1.08)	1.01 (0.90–1.12)	0.921
		High^d^	733/1576	0.75 (0.42–1.32)	0.94 (0.49–1.68)	0.844
	Penicillins	Intermediary ^c^	1295/3068	0.97 (0.88–1.07)	0.99 (0.88–1.10)	0.813
		High^d^	479/1748	0.69 (0.33–1.45)	0.87 (0.41–1.85)	0.717
	Macrolides	Intermediary ^c^	270/3635	1.21 (0.97–1.51)	1.28 (1.01–1.64)	0.041
		High^d^	129/1927	1.85 (0.76–4.52)	2.25 (0.90–5.65)	0.083
	Sulfonamides	Intermediary ^c^	136/3705	0.93 (0.68–1.28)	0.88 (0.62–1.26)	0.497
		High^d^	74/1958	0.64 (0.09–4.56)	0.64 (0.09–4.47)	0.618

**Table 3:** C-aAb as predictors of over-the-counter prescriptions within 1 year – men

C-aAb	Prescription	C-aAb level	C-aAb	Prescription	C-aAb level	C-aAb
IFNα	Antibacterials	Intermediary ^c^	905/3652	0.91 (0.81–1.03)	0.87 (0.76–1.00)	0.050
		High^d^	527/2001	1.12 (0.62–1.95)	1.08 (0.58–1.98)	0.810
	Penicillins	Intermediary ^c^	905/3810	0.91 (0.78–1.07)	0.89 (0.76–1.05)	0.057
		High^d^	388/2373	0.88 (0.39–1.96)	0.78 (0.36–1.78)	0.659
	Macrolides	Intermediary ^c^	258/3990	0.78 (0.62–1.01))	0.80 (0.62–1.04)	0.096
		High^d^	158/2192	0.97 (0.31–3.04)	1.17 (0.36–3.76)	0.941
GM-CSF	Antibacterials	Intermediary ^c^	874/3510	1.01 (0.84–1.21)	0.89 (0.71–1.10)	0.286
		High^d^	768 /3076	0.71 (0.34–1.49)	0.78 (0.30–1.66)	0.530
	Penicillins	Intermediary ^c^	874/3663	1.01 (0.83–1.21)	0.88 (0.71–1.10)	0.282
		High^d^	535/3209	0.59 (0.22–1.58)	0.53 (0.20–1.58)	0.286
	Macrolides	Intermediary ^c^	241/3842	1.16 (0.83–1.63)	0.99 (0.67–1.49)	0.991
		High^d^	211/3374	1.15 (0.37–3.61)	1.22 (0.39–3.87)	0.760
IL-10	Antibacterials	Intermediary ^c^	893/3602	1.01 (0.89–1.14)	0.99 (0.87–1.14)	0.921
		High^d^	468/1871	0.82 (0.41–1.64)	0.91 (0.39–1.85)	0.810
	Penicillins	Intermediary ^c^	893/3757	0.99 (0.88–1.13)	1.01 (0.88–1.14)	0.979
		High^d^	330/1950	0.93 (0.42–2.08)	0.92 (0.41–2.33)	0.930
	Macrolides	Intermediary ^c^	251/3937	0.95 (0.76–1.21)	0.92 (0.71–1.18)	0.511
		High^d^	133/2047	0.35 (0.05–2.52)	0.31 (0.04–2.40)	0.274
IL-6	Antibacterials	Intermediary ^c^	897/3628	0.99 (0.87–1.14)	1.02 (0.88–1.18)	0.770
		High^d^	302/1214	1.49 (0.87–2.51)	1.34 (0.75–2.42)	0.316
	Penicillins	Intermediary ^c^	897/3788	0.98 (0.86–1.12)	1.01 (0.88–1.17)	0.842
		High^d^	219/1261	1.19 (0.60–2.40)	1.14 (0.57–2.31)	0.822
	Macrolides	Intermediary ^c^	254/3963	0.97 (0.76–1.25)	0.94 (0.72–1.23)	0.656
		High^d^	87/1325	1.03 (0.33–3.24)	1.44 (0.45–4.65)	0.534
IL-1α	Antibacterials	Intermediary ^c^	905/3647	1.04 (0.92–1.19)	1.05 (0.91–1.21)	0.488
		High^d^	417/1737	0.57 (0.26–1.27)	0.48 (0.21–1.07)	0.086
	Penicillins	Intermediary ^c^	905/3805	1.04 (0.92–1.19)	1.06 (0.92–1.22)	0.385
		High^d^	288/1809	0.72 (0.30–1.73)	0.61 (0.23–1.57)	0.329
	Macrolides	Intermediary ^c^	256/3985	0.84 (0.67–1.07)	0.85 (0.65–1.10)	0.215
		High^d^	131/1887	0.98 (0.34–3.09)	0.86 (0.32–2.85)	0.814

^a^*IR* incidence rate per observed person-years, for adjusted analyses, *N* number of cases for adjusted analyses ^b^

^b^Cox regressions were performed using intermediary/high levels of c-aAb vs low levels as predictors vs low c-aAb levels and adjusted for age, smoking, BMI, combined oral contraceptives, and 1-year prescription history prior to c-aAb measurement/DBDS inclusion

^c^Intermediary c-aAb levels (negative control + 4SD < MFI < 99th percentile)

^d^High c-aAb levels (MFI > 99th percentile)

In men, high c-aAb levels did not predict prescription of any antimicrobials (Table 2).

In both men and women, high c-aAb levels neither predicted risk of antimycotic, tetracycline, nor antiviral prescriptions or infection-related diagnoses (data not shown).

### Cumulative c-aAb Incidence and Antimicrobial Prescriptions

**Fig. 2:**
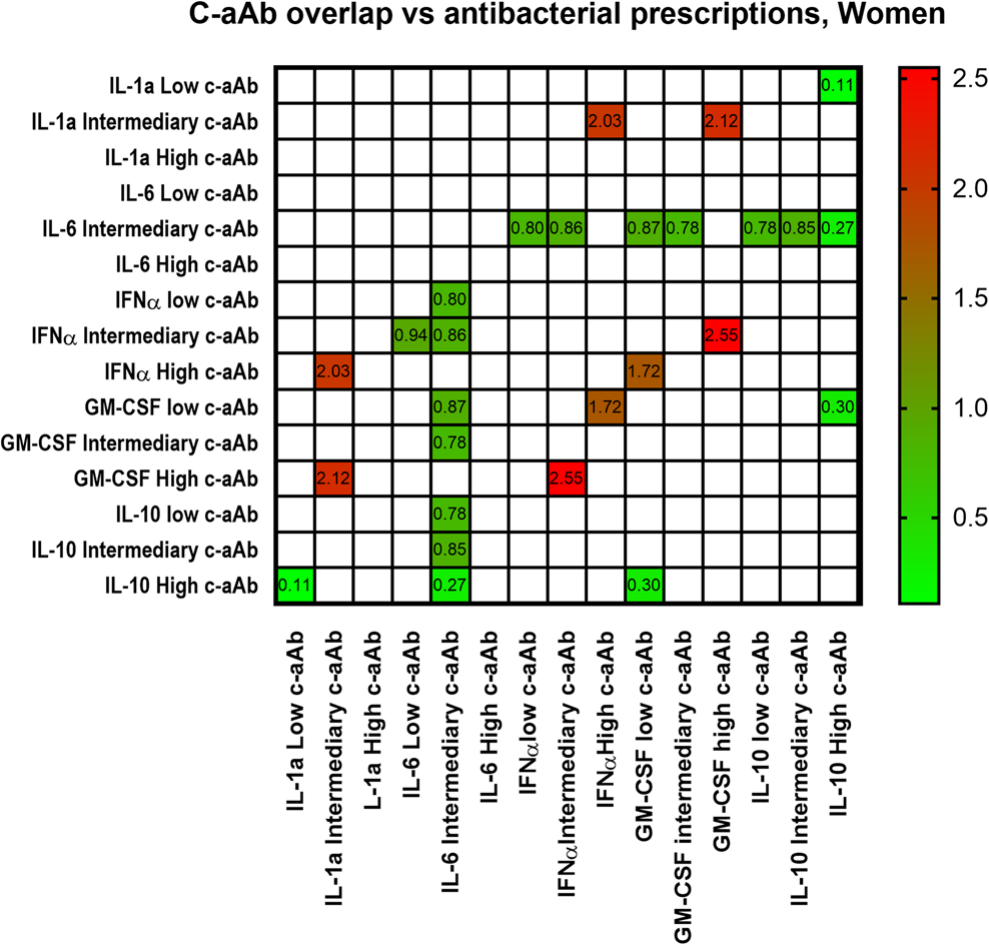
C-aAb overlap vs antimicrobial prescriptions in women Multivariate Cox proportional hazards models were used to investigate the intersection of low/intermediary and high levels of IL-1α, GM-CSF, IL-6, IFNα, and IL-10 c-aAb as predictors of risk of filing antimicrobial prescriptions within 1 year of c-aAb measurement. Analyses were performed for the interaction of two c-aAb at a time, with HR calculated using participants with low levels of both investigated c-aAb as a baseline. The models were adjusted for age, high levels of c-aAb, oral contraceptives, smoking, BMI, and 1 year prescription history of the prescription subtype in question prior to c-aAb measurement. For binary variables, high levels of c-aAb, usage of contraceptives, and having had at least one prescription a year before c-aAb measurement were coded as 1. High levels of c-aAb were defined as MFI above the 99th percentile, intermediary c-aAb levels were defined as MFI values > negative control + 4SD and < the 99th percentile, and low levels were defined as MFI values < negative control + 4SD. Data are presented as HR. All displayed HR corresponds to *p* values of < 0.05

In women, a pattern emerged where high levels of IL-10 c-aAb when combined with the presence of different levels of all the other c-aAb remained associated with a lower predicted risk of antibacterial prescriptions, particularly when IL-1α and GM-CSF c-aAb levels were low (Fig. 2). The low hazard ratio (HR) for antibacterial prescriptions induced by high levels of IL-10 c-aAb was further reduced when IL-10 c-aAb were combined with intermediary levels of IL-6 c-aAb (Fig. 2), whereas intermediary levels of IL-6 c-aAb combined with low or intermediary levels of other c-aAb (e.g., IFNα and GM-CSF c-aAb) were associated with HR < 1. Women with high levels of IFNα and GM-CSF c-aAb combined with intermediary levels of IL-1α, IFNα, or GM-CSF displayed the highest HR for antibacterial prescriptions indicating an additive biologic effect of the c-aAb.

### Cytokine Autoantibodies and Self-Reported Mental and Physical Health

**Fig. 3:**
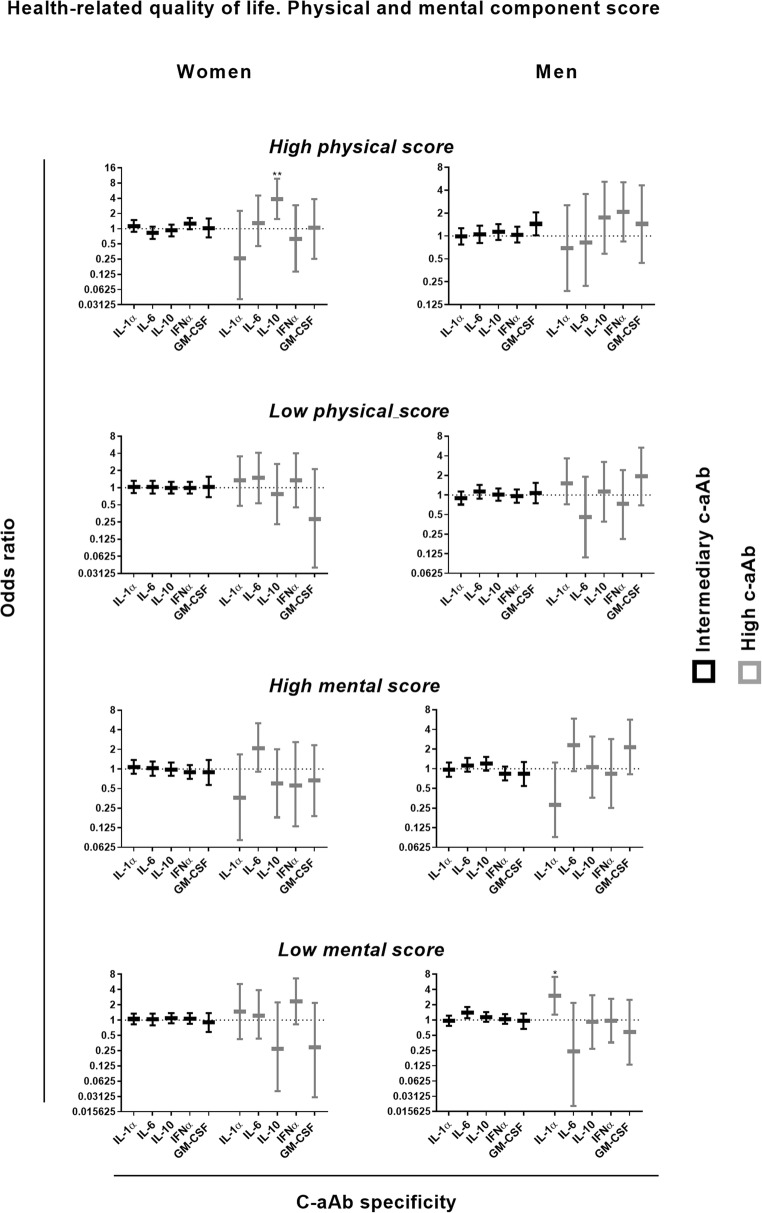
C-aAb as predictors of high/low self-reported physical/mental health Multivariate logistic regression models were used to investigate high levels c-aAb as predictors of having high or low scores of PCS and MCS. Independent variables include age, high levels of c-aAb, smoking, BMI, oral contraceptives, and 1 year prescription history of antibacterials. For binary variables, having high levels of c-aAb, usage of contraceptives, and having had at least one prescription a year before c-aAb measurement were coded as 1. High and low levels of PCS and MCS were defined as scores above or below the 90th and 10th percentiles, respectively. Intermediary c-aAb levels were defined as MFI values > negative control + 4SD and < the 99th percentile. High levels of c-aAb were defined as MFI above the 99th percentile with all high/low binary variables calculated separately for women and men. Data are presented as OR with 95% Confidence Interval (CI). * denotes a *p* value of < 0.05

In women, high levels of IL-10 c-aAb predicted a high (better) physical component score (PCS) (above the 90th percentile) (Fig. 3A, odds ratio (OR) = 3.87, 95% CI = 1.54–9.72, *p* = 0.004).

In men, high levels of IL-1α c-aAb predicted a lower (poorer) self-reported mental component score (MCS) (below the 10th percentile) in the adjusted models (OR = 3.02, 95% CI = 1.29–7.05, *p* = 0.010).

IL-6, IFNα, and GM-CSF, c-aAb were not associated with MCS or PCS for both men and women.

## Discussion

Cytokine autoantibodies are critical players in both immunodeficiencies and autoimmune disease, inflicting a variety of pro- or anti-inflammatory effects, depending on the context and their specificity. The present study investigated the association between different c-aAb directed against pro- and anti-inflammatory cytokines in healthy individuals and infectious risk, assessed as time until the next prescription of antimicrobials. We found that high-titer levels of c-aAb against pro- and anti-inflammatory cytokines predicted increased and reduced risk, respectively, of prescriptions of various antimicrobials as a proxy for infectious risk. As we observed positive associations between the c-aAb MFI signals, we also studied the pairwise association of c-aAb with prescriptions, which suggested a pattern of cumulative c-aAb effects. These findings suggest that c-aAb in healthy individuals are present in levels that may have immunomodulatory effects, and in the following, we speculate on the possible mechanisms of these.

In line with prior studies [16], we found c-aAb levels to be associated with sex and age, as well as a novel association of IL-6 c-aAb and oral contraceptives. As such, we observed a strong sex interaction for c-aAb and our investigated outcomes. In women, high levels of IFNα c-aAb were associated with increased risk of macrolide, sulfonamide, and antibacterial prescriptions in general. Previous studies have associated IFNα c-aAb levels with the occurrence of viral infections and have established a strong genetic component for the occurrence of this c-aAb [26–29]. This makes it a prime candidate for further studies as a risk factor for infection. The finding in the present study that INFα c-aAb levels associate with various antimicrobial prescriptions (indicative of different infections, i.e., respiratory infections and urinary tract infections) indicates a broader biologic effect of IFNα c-aAb though causality remains to be established. GM-CSF c-aAb have also been associated with multiple variants of opportunistic infections [30], and here we observed high levels of c-aAb to be associated with an increased risk of sulfonamide prescriptions in women. Interestingly, despite the previously observed association between IL-6 c-aAb and low levels of CRP [16], high levels of IL-6 c-aAb did not predict higher or lower risk of filing prescriptions in this study, which may reflect the pleiotropic roles of IL-6 as both a pro- and anti-inflammatory cytokine [31]. Furthermore, c-aAb have in some reports been observed to act as agonists of their target cytokine function [32], adding further complexity to our findings.

Notably, high levels of IL-10 c-aAb were associated with lower risk of penicillin and general antibacterial prescriptions in women, also when combined with other c-aAb. It may be speculated that the supposedly protective effects of IL-10 c-aAb may be due to suppression of the anti-inflammatory regulator IL-10, hereby creating a more pro-inflammatory and more potent antimicrobial environment, as previously reported [3].

As an alternative outcome to approximate c-aAb influence on participant health, we investigated the association to self-reported PCS and MCS health scores, hypothesizing that participants with c-aAb-mediated immunomodulation may present with lower or higher scores. Notably, we observed the same pattern for self-reported health scores as we did for antibacterial prescriptions, so women with a high level of IL-10 c-aAb had increased odds of high (good) PCS scores, again suggesting a potentially protective and positive effect of IL-10 c-aAb. It should be emphasized that the effects of IL-10 c-aAb are likely highly context-specific, as another previous study from our research group reported that IL-10 c-aAb were associated with increased risk of major adverse cardiovascular events (MACE) in kidney-transplanted patients, an outcome that is intrinsically linked to a pro-inflammatory environment [33]. Thus, though protective in regard to infections in healthy individuals, IL-10 c-aAb may constitute a risk factor for patients in whom excess inflammation may tip a balance towards adverse events such as organ transplant patients and autoimmune disease in general, again due to the cytokines’ many immunoregulatory functions [34, 35]. Conversely, c-aAb against pro-inflammatory cytokines IFNα and IL-1α have been linked to improved prognoses in autoimmune disease [6].

The observed associations between IL-10 c-aAb, prescriptions, and self-reported health being specific to women are notable, considering that women in our study had lower levels of detectable IL-10 c-aAb compared to men [16]. This finding may suggest a higher tolerance for IL-10 c-aAb in men. Conversely, we observed an association between high levels of IL-1α c-aAb and a low (poor) MCS in men, indicating increased sensitivity for this c-aAb in men. It should be noted that the significant effects were only observed for the highest levels of c-aAb, suggesting that these titers are a requirement for possible functional cytokine suppression and ensuing biologic effects.

The limited association between several high-titer c-aAb and infection or health-related outcomes may emphasize the redundancy of the human cytokine network, as c-aAb-mediated functional inhibition of just one cytokine may not be enough to challenge immune competence. Though there was a positive association between c-aAb, this seemed limited to their intermediary levels at most, with a near-total lack of association between high-titer c-aAb in individuals. This combined with their rarity means that we were unable to investigate cumulative incidences of high c-aAb levels as predictors of infection risk in this study. However, we assessed c-aAb overlap between the low/intermediary/high levels, and in women, we observed distinct patterns of higher or lower predicted risk of antibacterial prescriptions. The presence of intermediary or high levels of c-aAb against anti-inflammatory cytokine IL-10 and pleiotropic IL-6 coincided with reduced risk of antibacterial prescriptions, which may again be due to promoting an increasingly pro-inflammatory environment. Conversely, participants with high/intermediary levels of pro-inflammatory specific c-aAb had increased risk of antimicrobial prescriptions, suggesting additive immunosuppression by presence of several c-aAb against pro-inflammatory cytokines. Together, these data suggest that there may be cumulative immunomodulatory effects of c-aAb, depending on the specificity of the high-titer c-aAb present.

Overall, the data from the present study suggest that high levels of c-aAb mediate target-specific immunomodulation in healthy individuals. This, along with the many studies demonstrating associations between c-aAb and opportunistic infections [6, 8, 28, 30], raises further concern for the influence of high levels of c-aAb in immunocompromised individuals. Future studies in such patients are warranted to determine the impact of high levels of c-aAb on the immune system and overall patient health, as the influence of c-aAb on patient outcome which may be very different in this context. Given that immunological profiling, including cytokine assessment in plasma and stimulated cell cultures, is increasingly used as a precision medicine approach to improve care of immunocompromised patients, the present study supports the notion that monitoring c-aAb may be a critical component of such a profile.

In terms of strengths and limitations, this is to our knowledge the first study that investigates the association between cytokine autoantibody levels and infection risk and general health in healthy individuals. Because the study is cross-sectional and population-based and because outcomes are collected systematically and through public registries, we believe there are no systematic biases that could have interfered with the internal validity of the study. Though the use of a healthy and highly characterized blood donor cohort minimizes potential confounding effects of chronic illness or comorbidity, the generalizability of the results might be reduced because blood donors are generally healthier and have a healthier lifestyle as compared with the general population [36], which may result in higher than average self-perceived health scores compared to the general population [37]. Furthermore, it is critical to emphasize that the results of the study are purely based on associations and not causal relationships, which should be taken into account when interpreting the results. The number of analyses performed may also create a risk for Type I errors through multiple testing. Finally, there is also the possibility of systemic biases in the case of a self-reported outcome such as BMI, where weight may be underreported.

## Conclusions

In conclusion, we demonstrated that high levels of c-aAb specific for several pro-inflammatory cytokines predicted increased risk of infection in healthy individuals, whereas high levels of c-aAb against anti-inflammatory IL-10 predicted lower infection risk, as well as increased self-reported physical health.

These findings indicate that c-aAb in healthy individuals are present in levels that may have immunomodulatory effects, depending on the specific c-aAb-targeted cytokine. Overall, the findings emphasize that high-titer cytokine autoantibodies are biologically active in healthy individuals.

## Electronic supplementary material

ESM 1 (DOCX 28 kb)

ESM 2 (DOCX 25 kb)

## Abbreviations

**BMI**: Body mass index
**C-aAb**: Cytokine-specific autoantibodies
**CI**: Confidence interval
**DBDS**: The Danish Blood Donor Study
**DNPR**: Danish National Prescription Registry
**GM-CSF**: Granulocyte-macrophage colony-stimulating factor
**HR**: Hazard ratio
**IL**: Interleukin
**IFN**: Interferon
**MCS**: Mental component score
**MFI**: Median fluorescence intensity
**OC**: Oral contraceptives
**OR**: Odds ratio
**PCS**: Physical component score
**SD**: Standard deviation
