# Supplementary material for: Cytokine Autoantibodies Are Associated with Infection Risk and Self-Perceived Health: Results from the Danish Blood Donor Study
Source: J Clin Immunol. 2020 Jan 15;40(2):367–77. doi: 10.1007/s10875-020-00744-3 (PMC7082412; doi:10.1007/s10875-020-00744-3)
Supplement: Supplementary file 1 — (DOCX 28 kb) [file 10875_2020_744_MOESM1_ESM.docx]

**Supplementary material**

| Table S1: Association of intermediary/high c-aAb levels with epidemiological variables | | | | | | | | | | | | | | | | | | |
| --- | --- | --- | --- | --- | --- | --- | --- | --- | --- | --- | --- | --- | --- | --- | --- | --- | --- | --- |
|  |  |  |  | Age^a^ | | | Current Smoker ^b^ | | | BMI^a^ | | | Oral contraceptives^b^ | | | 1 year prescription history^c^ | | |
|  |  |  | Total N | N | Years | P | n | % Yes | P | n | BMI | p | N | % yes | P | N | % yes | P |
| IL-1α | Women | Low^d^ | 2,214 | 2,214 | 39.54 (12.64) |  | 2,054 | 18.79 |  | 2,074 | 24.45 (4.04) |  | 2,067 | 27.29 |  | 2,214 | 36.54 |  |
|  |  | Intermediary^e^ | 2,035 | 2,035 | 37.63 (12.02) | 0.0000 | 1,887 | 17.12 | 0.171 | 1,902 | 24.49 (4.19) | 0.7470 | 1,904 | 28.05 | 0.593 | 2,035 | 36.12 | 0.933 |
|  |  | High^f^ | 42 | 42 | 45.31 (11.62) | **0.0033** | 40 | 10.00 | 0.157 | 40 | 25.11 (3.75) | 0.3091 | 40 | 25.00 | 0.748 | 42 | 28.57 | 0.572 |
|  | Men | Low^d^ | 2,128 | 2,128 | 41.44 (12.22) |  | 1,949 | 17.09 |  | 1.954 | 25.62 (3.40) |  | - | - | - | 2,128 | 18.14 |  |
|  |  | Intermediary^e^ | 2,502 | 2,502 | 40.52 (12.19) | 0.0107 | 2,283 | 15.81 | 0.265 | 2,296 | 25.56 (3.47) | 0.5847 | - | - | - | 2,502 | 17.87 | 0.809 |
|  |  | High^f^ | 46 | 46 | 48.44 (10.42) | **0.0001** | 44 | 13.64 | 0.547 | 44 | 26.43 (3.70) | 0.1173 | - | - | - | 46 | 34.78 | **0.004** |
| IL-6 | Women | Low^d^ | 1,344 | 1,344 | 40.18 (13.15) |  | 1,240 | 19.35 |  | 1,254 | 24.56 (4.09) |  | 1,253 | 27.53 |  | 1,344 | 32.74 |  |
|  |  | Intermediary^e^ | 2,905 | 2,905 | 37.92 (11.97) | 0.0000 | 2,702 | 17.25 | 0.109 | 2,722 | 24.44 (4.12) | 0.3652 | 2,719 | 27.91 | 0.803 | 2,905 | 32.53 | 0.893 |
|  |  | High^f^ | 42 | 42 | 44.12 (19.54) | 0.0551 | 39 | 17.95 | 0.827 | 40 | 24.63 (4.59) | 0.9199 | 39 | 10.26 | **0.017** | 42 | 33.33 | 0.935 |
|  | Men | Low^d^ | 1,492 | 1,492 | 42.65 (12.54) |  | 1,370 | 18.98 |  | 1,375 | 25.75 (3.47) |  | - | - | - | 1,492 | 18.23 |  |
|  |  | Intermediary^e^ | 3,138 | 3,138 | 40.20 (11.98) | 0.0000 | 2,863 | 14.98 | 0.001 | 2,876 | 25.50 (3.40) | 0.0255 | - | - | - | 3,138 | 18.13 | 0.936 |
|  |  | High^f^ | 46 | 46 | 43.17 (12.74) | 0.7789 | 43 | 25.58 | 0.279 | 43 | 26.39 (4.45) | 0.2433 | - | - | - | 46 | 17.39 | 0.884 |
| IL-10 | Women | Low^d^ | 2,474 | 2,474 | 40.01 (12.79) |  | 2,291 | 19.60 |  | 2,315 | 24.51 (4.07) |  | 2,311 | 25.79 |  | 2,474 | 32.34 |  |
|  |  | Intermediary^e^ | 1,775 | 1,775 | 36.73 (11.54) | 0.0000 | 1,651 | 15.81 | 0.002 | 1,662 | 24.43 (4.16) | 0.5256 | 1,662 | 30.51 | 0.001 | 1,775 | 32.90 | 0.698 |
|  |  | High^f^ | 42 | 42 | 43.92 (11.74) | **0.0489** | 39 | 7.69 | 0.062 | 39 | 24.55 (4.62) | 0.9550 | 38 | 13.16 | 0.077 | 42 | 35.71 | 0.643 |
|  | Men | Low^d^ | 2,381 | 2,381 | 42.35 (12.53 |  | 2,171 | 18.64 |  | 2,178 | 25.59 (3.43 |  | - | - | - | 2,381 | 18.06 |  |
|  |  | Intermediary^e^ | 2,250 | 2,250 | 39.53 (11.69) | 0.0000 | 2,063 | 13.72 | 0.000 | 2,074 | 25.61 (3.46) | 0.8519 | - | - | - | 2,250 | 18.22 | 0..886 |
|  |  | High^f^ | 45 | 45 | 44.56 (12.57) | 0.2423 | 42 | 19.05 | 0.973 | 42 | 25.33 (2.66) | 0.6320 | - | - | - | 45 | 20.00 | 0.738 |
| IFNα | Women | Low^d^ | 2,322 | 2,322 | 40.34 (12.93) |  | 2,167 | 16.21 |  | 2,188 | 24.58 (4.12) |  | 2,189 | 25.90 |  | 2,322 | 32.77 |  |
|  |  | Intermediary^e^ | 1,927 | 1,927 | 26.69 (11.43) | 0.0000 | 1,777 | 13.51 | 0.010 | 1,789 | 24.34 (4.10) | 0,0720 | 1,783 | 29.61 | 0.009 | 1,927 | 32.33 | 0.759 |
|  |  | High^f^ | 42 | 42 | 38.92 (19.94) | 0.4804 | 37 | 16.37 | 0.370 | 39 | 24.69 (4.03) | 0,8647 | 39 | 33.33 | 0.295 | 42 | 35.71 | 0.688 |
|  | Men | Low^d^ | 2,520 | 2,520 | 42.55 (12.52) |  | 2,301 | 17.82 |  | 2,311 | 25.59 (3.37) |  | - | - | - | 2,520 | 18.13 |  |
|  |  | Intermediary^e^ | 2,110 | 2,110 | 39.19 (11.59) | 0.0000 | 1,931 | 14.76 | 0.007 | 1,939 | 25.62 (3.50) | 0.8555 | - | - | - | 2,110 | 18.10 | 0.978 |
|  |  | High^f^ | 46 | 46 | 39.96 (11.73) | 0.1631 | 44 | 11.36 | 0.266 | 44 | 25.03 (3.96) | 0.2764 | - | - | - | 46 | 21.74 | 0.530 |
| GM-CSF | Women | Low^d^ | 3.792 | 3.792 | 38.85 (12.42) |  | 3,518 | 18.16 |  | 3,549 | 25.60 (3.40) |  | 962 | 27.14 |  | 3.792 | 32.36 |  |
|  |  | Intermediary^e^ | 457 | 457 | 37.07 (12.16) | 0.0038 | 425 | 15.76 | 0.223 | 428 | 25.54 (3.56) | 0.3950 | 428 | 31.31 | 0.069 | 457 | 34.14 | 0.443 |
|  |  | High^f^ | 42 | 42 | 42.24 (11.17) | 0.0779 | 38 | 18.42 | 0.967 | 39 | 25.73 (4.51) | 0.6089 | 39 | 30.77 | 0.613 | 42 | 38.10 | 0.430 |
|  | Men | Low^d^ | 4,034 | 4,034 | 41.11 (12.23) |  | 3,686 | 17.09 |  | 3,700 | 25.60 (3.40) |  | - | - | - | 4,034 | 18.15 |  |
|  |  | Intermediary^e^ | 596 | 596 | 40.07 (11.90) | 0.0517 | 551 | 12.16 | 0.004 | 554 | 25.54 (3.57) | 0.7297 | - | - | - | 596 | 18.62 | 0.778 |
|  |  | High^f^ | 46 | 46 | 44.19 (14.16) | 0.0911 | 39 | 7.69 | 0.120 | 40 | 25.73 (4.51) | 0.8063 | - | - | - | 46 | 13.04 | 0.371 |

Chi-squared tests were used for analysis of binary variables. Participants with intermediary and high c-aAb levels were again separately compared to participants with low c-aAb levels.

a) T-tests were used for analysis of continuous variable correlation with intermediary/high c-aAb levels vs low c-aAb levels. Data presented as mean +SD

b) Chi-squared tests were used for analysis of binary variables. Participants with intermediary and high c-aAb levels were again separately compared to participants with low c-aAb levels. Data presented as percentage of population; current smokers and users of oral contraceptives coded as 0/1.

c) Analyses performed and presented as for b). Prescription history coded as having filed an antibacterial prescription within one year prior to c-aAb measurement/DBDS inclusion.

d) Low c-aAb levels (MFI < negative control + 4SD)

e) Intermediary c-aAb levels (negative control + 4SD < MFI <99^th^ percentile)

f) High c-aAb levels (MFI > 99^th^ percentile)
