# Supplementary material for: Cytokine Autoantibodies Are Associated with Infection Risk and Self-Perceived Health: Results from the Danish Blood Donor Study
Source: J Clin Immunol. 2020 Jan 15;40(2):367–77. doi: 10.1007/s10875-020-00744-3 (PMC7082412; doi:10.1007/s10875-020-00744-3)
Supplement: Supplementary file 2 — (DOCX 25 kb) [file 10875_2020_744_MOESM2_ESM.docx]

| **Table S2a: Associations between C-aAb MFI, women** | | | | | | | | | | | |
| --- | --- | --- | --- | --- | --- | --- | --- | --- | --- | --- | --- |
|  | | IL-1α c-aAb | | IL-6 c-aAb | | IL-10 c-aAb | | IFNα c-aAb | | GM-CSF c-aAb | |
|  |  | Test statistic (n) | P value | Test statistic (n) | P value | Test statistic (n) | P value | Test statistic (n) | P value | Test statistic (n) | P value |
| IL-1α c-aAb | Continuous variable^a^ |  |  | 0.28 | <0.0001 | 0.49 | <0.0001 | 0.37 | <0.0001 | 0.45 | <0.0001 |
|  | Ordinal variable A ^b^ |  |  | 300.84 | <0.001 | 689.40 | <0.001 | 341.83 | <0.001 | 85.16 | <0.001 |
|  | High vs non-high levels^c^ |  |  | 0.44  (0/4.291) | 0.507 | 0.77  (1/4.291) | 0.381 | 0.44  (0/4.291) | 0.507 | 0.77  (1/4.291) | 0.381 |
| IL-6 c-aAb | Continuous variable |  |  |  |  | 0.30 | <0.0001 | 0.25 | <0.0001 | 0.27 | <0.0001 |
|  | Ordinal variable |  |  |  |  | 306.89 | <0.001 | 192.23 | <0.001 | 25.33 | <0.001 |
|  | High vs non-high levels |  |  |  |  | 0.44  (0/4.291) | 0.507 | 0.77  (1/4.291) | 0.381 | 0.44  (0/4.291) | 0.507 |
| IL-10 c-aAb | Continuous variable |  |  |  |  |  |  | 0.44 | <0.0001 | 0.54 | <0.0001 |
|  | Ordinal variable |  |  |  |  |  |  | 480.97 | <0.001 | 72.09 | <0.001 |
|  | High vs non-high levels |  |  |  |  |  |  | 0.44  (0/4.291) | 0.507 | 0.44  (0/4.291) | 0.507 |
| IFNα c-aAb | Continuous variable |  |  |  |  |  |  |  |  | 0.43 | <0.0001 |
|  | Ordinal variable |  |  |  |  |  |  |  |  | 84.23 | <0.001 |
|  | High vs non-high levels |  |  |  |  |  |  |  |  | 0.44  (0/4.291) | 0.507 |
| GM-CSF c-aAb | Continuous variable |  |  |  |  |  |  |  |  |  |  |
|  | Ordinal variable |  |  |  |  |  |  |  |  |  |  |
|  | High vs non-high levels |  |  |  |  |  |  |  |  |  |  |

1. Spearman rank correlation of non-parametric c-aAb MFI signals. Results presented as speaman’s rho coefficient.
2. Chi-squared correlation of ordinal c-aAb variables where 2 = high levels of c-aAb (above the 99^th^ percentile MFI), 1 = intermediary levels of c-aAb (negative control + 4SD < MFI <99^th^ percentile) and 0 = c-aAb below the intermediary level
3. Chi-squared correlation of binary c-aAb variables, where 1 = high levels of c-aAb (above the 99^th^ percentile MFI) and 0 = c-aAb MFI below the high level. (n) indicates the overlap in high-positive individuals for this sex.

| **Table S2b: Associations between C-aAb MFI, men** | | | | | | | | | | | |
| --- | --- | --- | --- | --- | --- | --- | --- | --- | --- | --- | --- |
|  | | IL-1α c-aAb | | IL-6 c-aAb | | IL-10 c-aAb | | IFNα c-aAb | | GM-CSF c-aAb | |
|  |  | Test statistic (n) | P value | Test statistic (n) | P value | Test statistic (n) | P value | Test statistic (n) | P value | Test statistic (n) | P value |
| IL-1α c-aAb | Continuous variable^a^ |  |  | 0.25 | <0.0001 | 0.41 | <0.0001 | 0.31 | <0.0001 | 0.36 | <0.0001 |
|  | Ordinal variable A ^b^ |  |  | 333.86 | <0.001 | 643.10 | <0.001 | 360.90 | <0.001 | 58.98 | <0.001 |
|  | High vs non-high levels^c^ |  |  | 0.68 (1/4.676) | 0.438 | 0.45  (1/4.676) | 0.438 | 0.68 (1/4.676) | 0.438 | 5.40 (2/4.676) | 0.022 |
| IL-6 c-aAb | Continuous variable |  |  |  |  | 0.32 | <0.0001 | 0.29 | <0.0001 | 0.29 | <0.0001 |
|  | Ordinal variable |  |  |  |  | 374.30 | <0.001 | 320.50 | <0.001 | 48.65 | <0.001 |
|  | High vs non-high levels |  |  |  |  | 0.48  (0/4.676) | 0. 487 | 0.48  (0/4.676) | 0. 487 | 0.47  (0/4.676) | 0.492 |
| IL-10 c-aAb | Continuous variable |  |  |  |  |  |  | 0.44 | <0.0001 | 0.52 | <0.0001 |
|  | Ordinal variable |  |  |  |  |  |  | 522.35 | <0.001 | 93.50 | <0.001 |
|  | High vs non-high levels |  |  |  |  |  |  | 0.48  (0/4.676) | 0. 487 | 0.47  (0/4.676) | 0.492 |
| IFNα c-aAb | Continuous variable |  |  |  |  |  |  |  |  | 0.43 | <0.0001 |
|  | Ordinal variable |  |  |  |  |  |  |  |  | 116.40 | <0.001 |
|  | High vs non-high levels |  |  |  |  |  |  |  |  | 0.64  (1/4.676) | 0.425 |
| GM-CSF c-aAb | Continuous variable |  |  |  |  |  |  |  |  |  |  |
|  | Ordinal variable |  |  |  |  |  |  |  |  |  |  |
|  | High vs non-high levels |  |  |  |  |  |  |  |  |  |  |

1. Spearman rank correlation of non-parametric c-aAb MFI signals. Results presented as speaman’s rho coefficient.
2. Chi-squared correlation of ordinal c-aAb variables where 2 = high levels of c-aAb (above the 99^th^ percentile MFI), 1 = intermediary levels of c-aAb (negative control + 4SD < MFI <99^th^ percentile) and 0 = c-aAb below the intermediary level
3. Chi-squared correlation of binary c-aAb variables, where 1 = high levels of c-aAb (above the 99^th^ percentile MFI) and 0 = c-aAb MFI below the high level. (n) indicates the overlap in high-positive individuals for this sex.
